# Abiotic Synthesis with the C-C Bond Formation in Ethanol from CO_2_ over (Cu,*M*)(O,S) Catalysts with *M* = Ni, Sn, and Co

**DOI:** 10.1038/s41598-017-10705-3

**Published:** 2017-08-30

**Authors:** Xiaoyun Chen, Hairus Abdullah, Dong-Hau Kuo, Hsiu-Ni Huang, Cheng-Chung Fang

**Affiliations:** 1 Department of Materials Science and Engineering, National Taiwan University of Science and Technology, Taipei, 10607 Taiwan; 2 College of Material Engineering, Fujian Agriculture and Forestry University, Fuzhou, 350002 China; 3 Instrument Center, Office of Research and Development, National Taiwan Normal University, Taipei City, 11677 Taiwan; 4 Departments of Emergency Medicine, National Taiwan University Hospital and National Taiwan University College of Medicine, Taipei, 100 Taiwan

## Abstract

We demonstrate copper-based (Cu,*M)*(O,S) oxysulfide catalysts with *M* = Ni, Sn, and Co for the abiotic chemical synthesis of ethanol (EtOH) with the C-C bond formation by passing carbon dioxide (CO_2_) through an aqueous dispersion bath at ambient environment. (Cu,*Ni*)(O,S) with 12.1% anion vacancies had the best EtOH yield, followed by (Cu,*Sn*)(O,S) and (Cu,*Co*)(O,S). The ethanol yield with 0.2 g (Cu,*Ni*)(O,S) catalyst over a span of 20 h achieved 5.2 mg. The ethanol yield is inversely proportional to the amount of anion vacancy. The kinetic mechanism for converting the dissolved CO_2_ into the C_2_ oxygenate is proposed. Molecular interaction, pinning, and bond weakening with anion vacancy of highly strained catalyst, the electron hopping at Cu^+^/Cu^2+^ sites, and the reaction orientation of hydrocarbon intermediates are the three critical issues in order to make the ambient chemical conversion of inorganic CO_2_ to organic EtOH with the C-C bond formation in water realized. On the other hand, Cu(O,S) with the highest amount of 22.7% anion vacancies did not produce ethanol due to its strain energy relaxation opposing to the pinning and weakening of O-H and C-O bonds.

## Introduction

CO_2_, the combustion by-product of the carbon-based oils used for human activities, has been a serious and worrying issue for its high concentration in atmosphere, which has led to the global climate change. Carbon management is a world-wide topic. Artificial photosynthesis^1^, a chemical reaction to mimic natural photosynthesis for the generation of renewable energy, has been described as “Chemistry’s Greatest Challenge”^2^. Catalysts for the conversion of CO_2_ include metal complex, metal oxide, sulfide, phosphide etc.^3–8^. At this stage, the synthesis of methanol (MeOH) *via* CO_2_ hydrogenation utilizes composite catalysts under a high power lamp^9–13^ or under high temperature/pressure^14–19^.

Ethanol from the CO_2_ conversion is much more difficult than MeOH due to the C-C chain formation. Wang *et al*. used Au/TiO_2_ catalyst to generate EtOH at 200 °C and 60 bar from CO_2_ and H_2_^20^ or from a reverse water-gas shift reaction, while most reactions were executed above 250 °C and 50 bar to produce higher alcohols with lower ethanol selectivity^21–25^. Chen, Choi, and Thompson proceeded CO_2_ hydrogenation in *liquid* 1,4-dioxane to MeOH as a major phase at 135 °C but the mixed hydrocarbons at 200 °C^26^. Song *et al*. demonstrated the electrochemical conversion of CO_2_ to ethanol with high efficiency at −1.2 V *vs* RHE^27^. To convert CO_2_ into C_1_-C_3_ oxygenates in the presence of noble metal-free catalyst without adding sacrificial electron donors or hole scavengers, and without stimulating with thermal, photo, or electrical energy has been quite difficult.

Natural photosynthesis in plants has set an adamant standard that sunlight is a necessity to drive the glucose-formation reaction at the ambient environment. Artificial photosynthesis is an approach for biomimetics. The abiotic synthesis of organic molecules has involved the spark discharge, the irradiation of ultraviolet light in the presence of mineral catalysts, the hydrothermal vents on the ocean floor, the impact energy when comets or asteroids struck the early earth. Abiotic chemical synthesis without the driving force of external inputs in heat, light, and electricity can be a challenge. A preliminary study on the CO_2_/MeOH/H_2_ cycle by (Cu,*Mn*)(O,S) nanoflowers had been presented^28^. Here with lattice strain energy to replace sunlight energy as the driving force to weaken chemical bonds, our aqueous (Cu,*M*)(O,S) catalyst system with *M* = Ni, Sn, and Co is demonstrated to produce EtOH from the dissolved CO_2_ and water with the C-C bond formation at normal temperature and pressure. The understanding on the C-C bond formation by such a thermodynamically difficult reaction to mimic photosynthesis can help in the catalyst design for converting inorganic into organic species.

For the catalyst preparation^28, 29^, 4.8 g cupric nitrate (Cu(NO_3_)_2_·2.5H_2_O) was added to a 500 ml deionized water solution, and then 3 g NiCl_2_·6H_2_O, SnCl_2_·*x*H_2_O, or CoCl_2_ for the second metal was mixed into the solution. After stirring for 30 min, 1.5 g thioacetamide (CH_3_CSNH_2_) was added into the mixed solution for another 30 min stirring. After heating to 90 °C, the precursor solution was adjusted with 0.3 ml hydrazine (N_2_H_4_). After 2 h heating at 90 °C, the reaction was complete and the precipitation solids, individually labeled as (Cu,*Ni*)(O,S), (Cu,*Sn*)(O,S), and (Cu,*Co*)(O,S), were washed, dried at 60 °C with a vacuum dryer, and collected. The precipitation solids were individually labeled as (Cu,*Ni*)(O,S), (Cu,*Sn*)(O,S), and (Cu,*Co*)(O,S). For comparison, Cu(O,S) was synthesized with the same procedure just without adding zinc acetate.

Surface composition and chemical state of the (Cu,*M*)(O,S) catalyst were investigated with XPS (VG Scientific ESCALAB 250) photoelectron spectrometry under the Al Kα X-rays (*hv = *1486.6 eV) radiation with carbon C1*s* (*E*a = 284.62 eV) for calibration. The particle size and morphology of the catalysts were examined by high resolution transmission electron microscopy (HR-TEM, H-7000, Hitachi). The crystal structure of samples was characterized by using X-ray diffraction analysis (Bruker D2 phaser, Japan) using Cu Kα radiation.

The conversion of CO_2_ by (Cu,*M*)(O,S) (*M* = Ni, Sn, and Co) was carried out in a home-made and jacketed quartz reactor. In the experiment, 0.2 g catalyst was added into the reactor with 70 mL distilled water, then CO_2_ gas was passed into the reactor by adding droplets of the HNO_3_ aqueous solution into the NaHCO_3_-dispersed solution. The amount of HNO_3_ added to NaHCO_3_-dispersed solution was controlled by automatic infusion machine with the flow rate of 0.5 mL/h. Prior to adding HNO_3_ to the solution, argon gas at 100 mL/min was flowed into the reactor to purge out all the atmospheric gases in reactor. The experiments were executed in a period of 20 h to collect sufficient amount of product to minimize the system errors including alcohol vaporization. The collected and centrifuged reaction solutions were analyzed by HP 6890 series gas chromatography equipped with HP 5973 mass selective detector, i.e. by GC-MS. During the experiment, the unreacted gas was collected in a sample bag for further GC analysis. Our experimental setup is schematically shown in Fig. S1 in Supporting Information.

**Figure 1:**
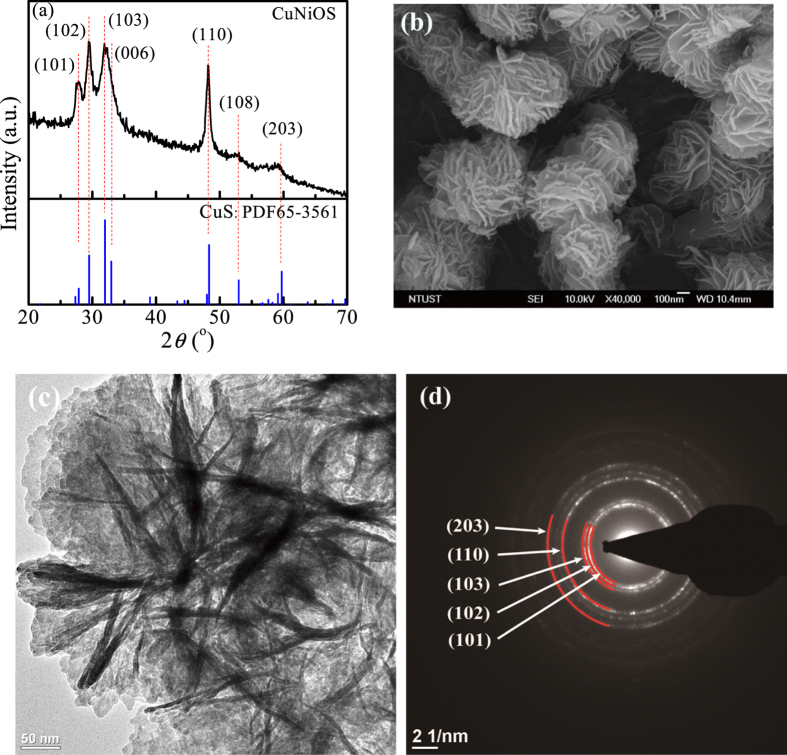
Microstructural and structural characterizations of (Cu,*Ni*)(O,S) catalyst: (**a**) XRD spectrum, (**b**) SEM image, (**c**) TEM image, and (**d**) SAED pattern.

Figure 1a shows the XRD diffraction pattern of (Cu,*Ni*)(O,S). The peaks of XRD pattern indicated that (Cu,*Ni*)(O,S) had a hexagonal CuS covellite structure in accordance with JCPDS No. 65-3561. The major peaks located at about 27.97, 29.51, 32.16, 32.98, 48.22, 52.93, and 59.64 corresponded to the (101), (102), (103), (006), (110), (108), and (203) crystal planes, respectively. No second phases were detected, which indicates the formation of solid solution. Figure 1b shows the FE-SEM image of (Cu,*Ni*)(O,S) with particle size in the range of 300~500 nm and the shape of petal-gathered flowers. Figure 1c shows the TEM image of (Cu,*Ni*)(O,S), which further verifies the (Cu,*Ni*)(O,S) microstructure. Figure 1d shows the selected area electron diffraction (SAED) pattern of (Cu,*Ni*)(O,S) catalyst. The ring patterns from the (101), (102), (103), (110), and (203) planes explain its polycrystalline nature. The ring patterns displayed a broad circular band instead of a sharp ring, which explains the solid solution nature in (Cu,*Ni*)(O,S). Similar analyses were shown for (Cu,*Sn*)(O,S) in Fig. S2, (Cu,*Co*)(O,S) in Fig. S3, and Cu(O,S) in Fig. S4.

**Figure 2:**
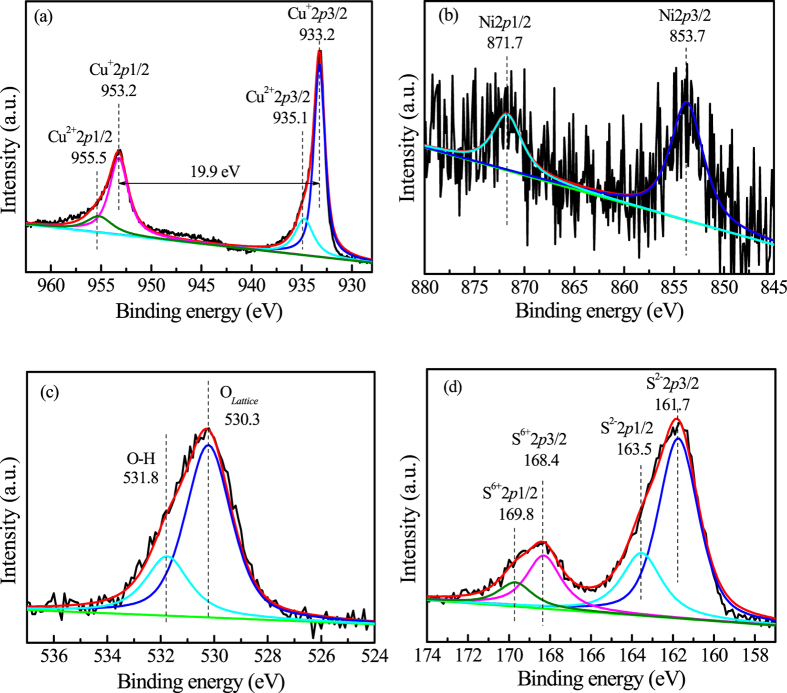
High resolution XPS spectra of (**a**) Cu2*p*, (**b**) Ni2*p*, (**c**) O1*s*, and (**d**) S2*p* for (Cu,*Ni*)(O,S).

Figure 2a shows the high resolution (HR) XPS spectrum of Cu2p. The asymmetric shape of the Cu2p peak represented the different chemical states of Cu in the (Cu,*Ni*)(O,S) catalyst. The two peak positions of 2p3/2 and 2p1/2 at 933.2 and 953.2 eV, respectively, with a peak separation of 20.0 eV due to the spin-orbit splitting indicated that copper was in the Cu^+^ state^30^. On the other hand, the two peak positions at 935.1 and 955.5 eV were to identify the monovalent Cu^2+^ ^31^. The Cu^+^ and Cu^2+^ molar contents were calculated to be 76.7% and 23.3%, respectively. The ratio between Cu^+^ and Cu^2+^ is about 3.29. Figure 2b shows HR-XPS spectra of Ni2p for (Cu,*Ni*)(O,S) catalyst. The two peak positions of 2p3/2 and 2p1/2 were observed at 853.7 and 871.7 eV, respectively, and were contributed from Ni^2+^ ^31^. Figure 2c shows the HR-XPS spectra of O1s for (Cu,*Ni*)(O,S) catalyst. The asymmetric shape of the O1s peak was attributed to the hydroxyl oxygen O-H (531.8 eV) and the lattice oxygen (530.3 eV)^32–35^. Figure 2d shows the HR-XPS spectra of S2p for (Cu,*Ni*)(O,S) catalyst. The peaks at 161.7 and 163.5 eV were attributed to S^−2^ and the peaks at 168.4 and 169.8 eV to S^6+^ ^36, 37^. The S^6+^ and S^2−^ molar contents were calculated to be 19.05% and 80.95%, respectively. Such a large amount of S^6+^ in ionically bonded materials is rare to see. Similar analyses were shown for (Cu,*Sn*)(O,S) in Fig. S2, (Cu,*Co*)(O,S) in Fig. S3, and Cu(O,S) in Fig. S4.

Table 1 summarizes the results of XPS element analyses of (Cu,*Ni*)(O,S), (Cu,*Sn*)(O,S), (Cu,*Co*)(O,S), and Cu(O,S) catalysts. All the compounds had the lattice oxygen/S^2−^ ratio of < 1.0, indicating they are sulfur-rich. As Cu has a much higher content than Ni, Sn, or Co, the (Cu,*M*)(O,S) is actually the Cu-based oxysulfide solid solution. During the catalyst preparation step, the amount of the Ni, Sn, or Co precursor was not little but their content in (Cu,*M*)(O,S) catalyst was much less, which indicates the difficulty in dissolving *M* into the Cu(O,S) lattice under our low-temperature process below 100 °C. To better show the complicated composition, the molecular formula of (Cu,*Ni*)(O,S) is listed as $(C{u}_{0.328}^{+}C{u}_{0.099}^{2+}{{\rm{Ni}}}_{0.016}^{2+}{S}_{0.054}^{6+})({O}_{0.206}^{2-}{S}_{0.231}^{2-})$. For covellite structure of CuS, the cation: anion ratio remains 1:1. Based upon the total cation site of 1, the above molecular formula can be re-written as $(C{u}_{0.660}^{+}C{u}_{0.199}^{2+}{{\rm{Ni}}}_{0.032}^{2+}{S}_{0.109}^{6+})({O}_{0.414}^{2-}{S}_{0.465}^{2-})$. The total anion content is 0.879. There is 12.1% anion vacancies in (Cu,*Ni*)(O,S). From the similar analyses, there were 13.7% anion vacancies for (Cu,*Sn*)(O,S), 15.1% for (Cu,*Co*)(O,S), and 22.7% for Cu(O,S). The basic trend is that Cu(O,S) with the lowest Cu^+^/Cu molar ratio of 0.755 shows the highest S^6+^/S^2−^ ratio of 0.264 and the highest anion vacancy ratio of 22.7%. The addition of the second metal like Ni, Sn, or Co apparently decreases the S^6+^ content and the anion vacancy ratio. Cu(O,S) has a much looser lattice structure due to the 22.7% anion vacancies.

**Table 1:** XPS composition analyses of (Cu,*M*)(O,S) catalysts with *M* = Ni, Sn, and Co.

Catalyst	Molar percentage				Cu molar percentage		O molar percentage		S molar percentage		S^6+^/ S^2−^ molar ratio	O in anion site (molar ratio)	Anion vacancy percentage
	Cu	*M*	O	S	Cu^+^	Cu^2+^	O-H	O_*Lattice*_	S^6+^	S^2−^			
Cu*Ni*OS	42.70	1.58	27.15	28.57	76.70	23.30	24.12	75.88	19.05	80.95	0.235	0.471	12.1
Cu*Sn*OS	41.42	4.37	25.71	28.50	75.6	24.4	25.4	74.6	15.3	84.7	0.181	0.443	13.7
Cu*Co*OS	43.1	2.0	23.1	31.8	78.9	21.1	26.2	73.8	17.9	82.1	0.218	0.395	15.1
CuOS	46.92	0	24.42	28.66	75.5	24.5	25.6	74.4	20.9	79.1	0.264	0.445	22.7

**Figure 3:**
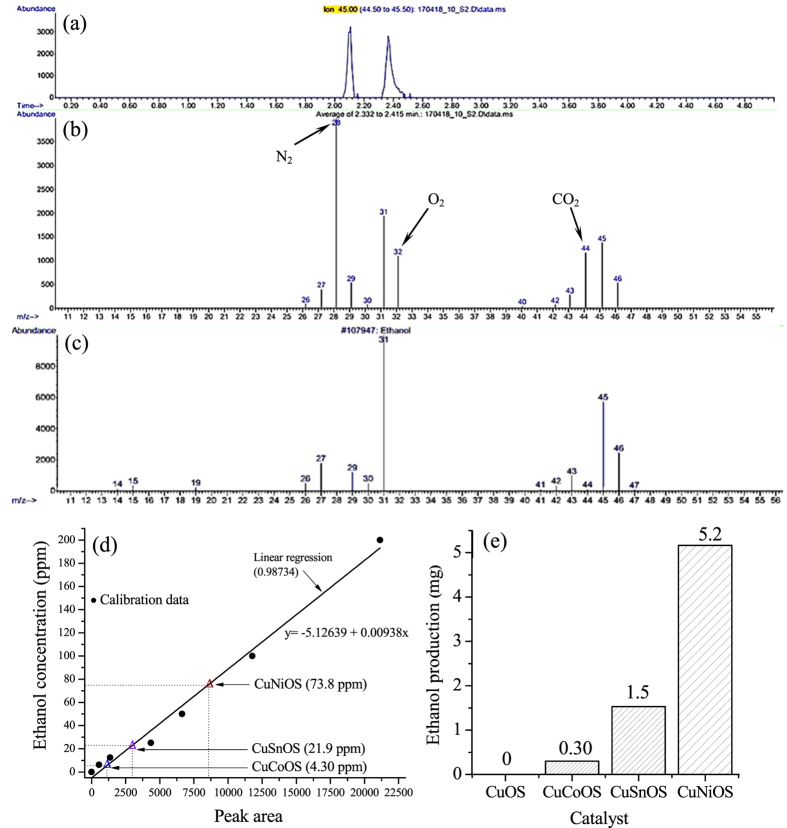
(**a**) Gas chromatogram of reaction solution catalyzed by (Cu,*Ni*)(O,S) with (**b**) the mass spectra in the retention time from 2.332 to 2.415 min. (**c**) The standard mass spectra of ethanol. (**d**) The ethanol yields in unit of ppm together with the calibration line. (**e**) Ethanol production in unit of mg for different catalysts over a period of 20 h.

Figure 3a shows gas chromatogram of reaction solution catalyzed by (Cu,*Ni*)(O,S). Figure 3b shows the mass spectra for the species in the retention time from 2.332 to 2.415 min, as compared with the standard mass spectra of ethanol in Fig. 3c. The first peak at the retention time of ~2.1 min was originated from the CO_2_ contribution. The mass spectra of our reaction solution were contributed from nitrogen, oxygen, and CO_2_ with the m/z ratios at 28, 32, and 44, respectively. Nitrogen and oxygen peaks were contributed from the air during the GC measurement. The CO_2_ peak indicates the existences of dissolved CO_2_ in solution. It is obvious that ethanol peaks in Fig. 3b match well to those in standard file. Other GC-MS data for (Cu,*Sn*)(O,S)- and (Cu,*Co*)(O,S)-catalyzed reaction solutions are provided in Figs S5 and S6, respectively. There was no reaction occur for Cu(O,S) in water with CO_2_. Figure 3d shows the ethanol yields in the unit of ppm with different catalysts together with the ethanol calibration line. The amounts of ethanol obtained with (Cu,*Ni*)(O,S), (Cu,*Sn*)(O,S) and (Cu,*Co*)(O,S) are 73.8, 21.9, and 4.3 ppm, respectively. Figure 3e shows the ethanol production with different catalysts over a span of 20 h. They are 5.2, 1.5, 0.30, and 0 mg for (Cu,*Ni*)(O,S), (Cu,*Sn*)(O,S), (Cu,*Co*)(O,S), and Cu(O,S), respectively.

(Cu,*Ni*)(O,S) with a good ethanol yield for the CO_2_ hydrogenation by flowing CO_2_ into the catalyst-dispersed aqueous solution shows several unique characteristics: (1) Cu^+^ is dominant over Cu^2+^ in the Cu^2+^S covellite structure with a ratio of Cu^+^/Cu of ~0.77, (2) there are 10.9% S^6+^ in cation and 12.1% anion vacancies, (3) the cation site contains four kinds of ions in Cu^+^, Cu^2+^, Ni^2+^, and S^6+^ and three kinds of valence charges in 1 + , 2 + , and 6 + , (4) the anion site is occupied with vacancy, O^2−^, and S^2−^, and (5) (Cu,*Ni*)(O,S) can generate EtOH, but it is not for Cu(O,S). As there are much more Cu^+^ cations than Cu^2+^ in the Cu^2+^S structure (Fig. 2a), anion vacancy and S^6+^ cation have to form in order to keep charge neutrality and to hold the covellite structure. The multiple cations in Cu^+^ of 0.77 Å in radius, Cu^2+^ of 0.73 Å, Ni^2+^ of 0.69 Å, and S^6+^ of only 0.29 Å and the multiple anions in O^2−^ of 1.40 Å and S^2−^ of 1.84 Å^38^ are the characters of (Cu,*Ni*)(O,S). Together with the high configuration entropy due to the complex composition, the highly distorted and heavily strained (Cu,*Ni*)(O,S) catalyst is at a high strain energy state and is much active. Figure S7 displays the high-resolution TEM image of (Cu,*Sn*)(O,S). In that figure, an enlarged image is to demonstrate the bent and distorted lattice structure of (Cu,*Sn*)(O,S). Figure S8 shows Raman spectra of (Cu,*M*)(O,S), Cu(O,S), and commercially available CuS. The flatten Raman spectra for self-made oxysulfides did not show any characteristic chemical bonding. The multiple cations and anions and the distorted lattice structure randomize the chemical bonding of catalysts with no characteristic peaks to show up. A covellite structure with a high entropy configuration is obtained.

The order in the ethanol yield, (Cu,*Ni*)(O,S) > (Cu,*Sn*)(O,S) > (Cu,*Co*)(O,S) >> Cu(O,S), does not show the correlations with the compositions in Table 1 and morphologies in Figs. 2b, S2b, and S3b, but shows a reverse relation with the order in the amount of anion vacancies: (Cu,*Ni*)(O,S) (12.1%) < (Cu,*Sn*)(O,S) (13.7%) < (Cu,*Co*)*(*O,S) (15.1%) < Cu(O,S) (22.7%). It is apparent that the ambient conversion of CO_2_ in water is strongly related to the stored strain energy in catalyst. Cu(O,S) releases its strain energy due to the 22.7% anion vacancies and cannot be used for chemical conversion. The best metal *M* in catalyst is the one to build inside the highest strain energy, which involves the optimization among the ionic size effect, the type and content of *M*, the equilibrium defect configuration, the charge state of cation etc.

**Figure 4:**
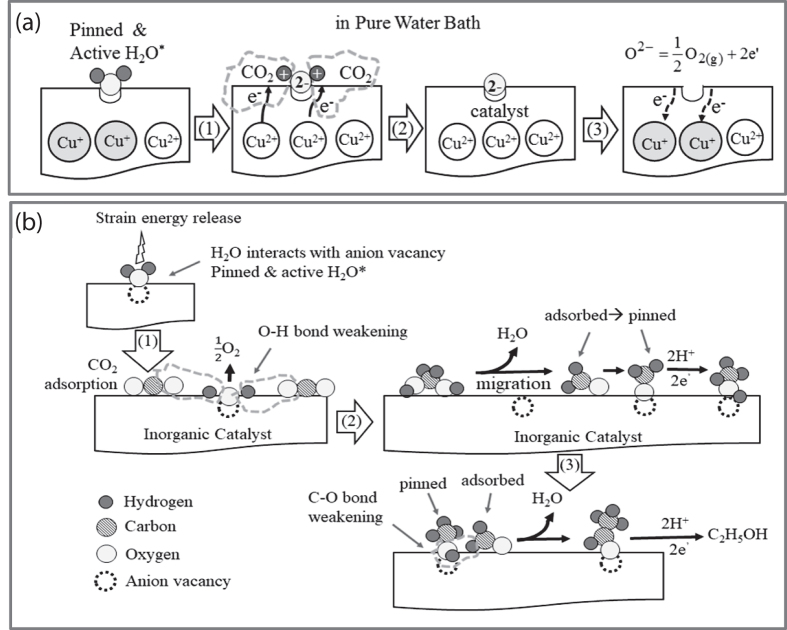
(**a**) The oxygen-exchangeable mechanism for the interaction between anion vacancy of catalyst and the solvent of water and (**b**) the kinetic mechanism for the formation of ethanol from CO_2_ in water.

Based upon the anion vacancy and the distorted and strained lattice in catalysts, two kinetic mechanisms are proposed for the ambient conversion of CO_2_ to EtOH with the C-C bond formation in water: the oxygen-exchangeable mechanism and the ethanol-forming kinetic mechanism, as shown in Fig. 4a,b, respectively. For the formation of EtOH from CO_2_, it needs the hydrogenation and the C-C bond formation. *The addition of hydrogen, which solely comes from water, for CO*_2_ *reduction has to happen*. Water needs to involve with the CO_2_-to-EtOH conversion. As our (Cu,*Ni*)(O,S) has its lattice highly distorted, each of its nanoparticle can be viewed as a “high energy nanobomb” with high strain energy. The anion vacancies of catalyst surrounding with cations prefer to trap the highly electronegative oxygen of H_2_O, simultaneously with the strain energy release from (Cu,*Ni*)(O,S) “nanobomb”. Therefore, H_2_O molecules are pinned at the anion vacancies of (Cu,*Ni*)(O,S) surfaces with the outward-pointed O-H bond weakened. This pinning-to-bond weakening step of H_2_O can be viewed as the activation step of reaction initiation. The H_2_O-pinning equation by anion vacancy of catalyst, V_anion(catal.)_, is shown in Eq. (1) to form the pinned and active H_2_O (H_2_O^*^):

1 $${{\rm{H}}}_{2}{{\rm{O}}}_{({\rm{aq}}.)}+{{\rm{V}}}_{\text{anion}(\text{catal}.)}\mathop{\longrightarrow }\limits^{\begin{array}{c}{\rm{Catalyst}}\\ \begin{array}{cc}{\rm{Strain}} & {\rm{Energy}}\end{array}\end{array}}\begin{array}{cc}{\rm{Active}} & {{\rm{H}}}_{2}{{\rm{O}}}_{{\rm{anion}}({\rm{catal}}.)}\end{array}$$

When CO_2_ starts to flow through the reactor, it always undergoes adsorption, migration, and reaction with the trapped and bond-weaken H_2_O*. Therefore, the next step after water trapping is the reaction between the in-coming and adsorbed CO_2_ and the trapped and weakened H_2_O* on catalyst surface, as shown in the step (1) in Fig. 4a,b. As the adsorbed CO_2_ reacts with the dissociated protons from H_2_O^*^, oxygen remains trapped at vacancy. For the charge neutrality consideration, the formation of each trapped oxygen needs to accompany with the transition of 2Cu^+^→ 2Cu^2+^  + 2e’ with the release of 2 electrons. The released electrons are required for the CO_2_ reduction reaction with protons. After CO_2_ reaction, the anion vacancy of catalyst covers with oxygen ion and the Cu^2+^ content increases, as shown in step (2) of Fig. 4a. The trapped O^2−^ can be released to form O_2_ *via* the equation of O^2−^ → $\frac{1}{2}$ O_2(g)_ + 2e’, simultaneously with the 2Cu^2+^  + 2e’ → 2Cu^+^ transition, as shown in step (3) of Fig. 4a. The transition from Cu^+^ to Cu^2+^ and its reverse one from Cu^2+^ to Cu^+^ (Fig. 4a) can re-install the Cu^+^/Cu^2+^ ratio back to its original charge state or the catalyst is refreshed back to its highly strain energy state. The refreshed and re-appeared anion vacancy will trap H_2_O again or to wait for the arrival of reaction intermediates, as discussed next.

On the CO_2_ reaction part, when it approaches the trapped and bond weakened H_2_O*, their molecules interact together to weaken and break two HO−H bonds in H_2_O* and to form CHO(OH) with the simultaneous assistance of 2e^−^ from the oxygen-exchangeable mechanism in Fig. 4a. CHO(OH) further converts to CH_2_(OH)_2_ with additional 2 H^+^ from the HO−H bond weakening and 2e’ from the Cu^+^/Cu^2+^ transition, and then to form the adsorbed formaldehyde (CH_2_O_(ad.)_) by de-hydrolysis with the liberation of one H_2_O molecule, as shown in step (2) of Fig. 4b. At this stage, the catalyst surface is covered with the “refreshed and empty” and the “H_2_O^*^-trapped” anion vacancies together with the adsorbed CH_2_O_(ad.)_, which is evaluated as the basic unit for natural saccharides with the general formula of (CH_2_O)_n_ from natural photosynthesis. The acetyl group of CH_3_CO^−^ was proposed as the intermediate for the formation of EtOH from the gas phase reaction between CO_2_ and H_2_^20^, while CH_*x*_O was recommended from the thermo-chemical reaction between CO and H_2_^21^. Some surface-diffusive CH_2_O_(ad.)_ molecules on catalyst surface in this work have their negatively charged ends of the C=O functional group trapped or pinned at the refreshed anion vacancies and then form active CH_3_OH* after hydrogenation. The trapped CH_3_OH* shows the weakened C-O bond due to the molecule pinning on catalyst. Some surface-diffusive CH_2_O_(ad.)_ molecules react with the pinned and weakened CH_3_OH* to form the desorbed C_2_H_5_OH, as shown in step (3) in Fig. 4b. Here the ethanol formation is proposed by the reaction between *the pinned and aligned* and *the adsorbed and movable* species. The formed and adsorbed ethanol loses its strong interaction with the anion vacancy of catalyst and is released into solvent. Therefore, there are no C_n_ species with n > 2.

Based upon the ability of catalyst in dynamically supplying protons and electrons, as shown in Fig. 4a, the formation reaction of ethanol is similar to the natural photosynthesis with the following reaction:

2 $${{\rm{2CO}}}_{2(\text{ad}.)}+{{\rm{12H}}}_{(\text{aq}.)}^{+}+{{\rm{12e}}}_{(\text{aq}.)}^{-}={{\rm{C}}}_{2}{{\rm{H}}}_{5}{{\rm{OH}}}_{(\text{aq}.)}+{{\rm{3H}}}_{2}{{\rm{O}}}_{(\text{aq}.)}$$

The driving force to overcome this reaction barrier is the “released strain energy” from catalyst instead of sunlight. Our net reaction equation is shown below:

3 $${{\rm{2CO}}}_{2(\text{ad}.)}+{{\rm{6H}}}_{2}{{\rm{O}}}_{\text{anion}(\text{catal}.)}^{\ast }\mathop{\longrightarrow }\limits^{\begin{array}{c}{\rm{Released}}\\ \begin{array}{cc}{\rm{Strain}} & {\rm{Energy}}\end{array}\end{array}}{{\rm{C}}}_{2}{{\rm{H}}}_{5}{{\rm{OH}}}_{(\text{aq}.)}+{{\rm{3O}}}_{2(g)}+{{\rm{3H}}}_{2}{{\rm{O}}}_{(\text{aq}.)}$$

With the aids of (a) the strain energy release, (b) the trapped and active H_2_O* with weakened O-H bonds to elevate its chemical potential above the standard Gibbs formation energy, and (c) the heat liberation during the interaction between the dissolved CO_2_ and the activated H_2_O^*^, the conversion of CO_2_ to EtOH in water is proceeded.

The CO_2_-to-EtOH conversion cannot proceed without electron transport. Therefore, we make the first proposal: *Catalyst with the feasibility to reversibly form the multiple charged cation states can benefit the electron hopping transport required for CO*_2_ *hydrogenation*. Saito *et al*. referred that the high activity in methanol synthesis over the Cu/ZnO-based catalyst by the thermo-conversion was related to the ratio of Cu^+^/Cu at ~0.7^39^, which is consistent with our ratio of 0.755–0.789. Lin and Frei observed the oxidation of Cu(I) at the CO_2_ photoreduction^40^.

Water being trapped and weakened over catalyst by anion vacancy are crucial to initiate the CO_2_ conversion reaction. The function of water in catalysis with inorganic oxides has been identified^41–43^. Therefore, we make the second proposal: *Catalyst with the adequate amount of anion vacancies and the higher oxygen-exchangeable ability with water due to the strain energy consideration can weakened the HO−H bonds in the trapped and active water for generating protons required in* Eq. (2). As anion vacancies are needed for ambient conversion, however, Cu(O,S) with a large amount of anion vacancies (22.7%) has a too loose structure instead of strained one to establish the sufficient strain energy and to initiate the HO−H bond trapping/weakening in active H_2_O*. J.C. Frost^44^ mentioned that the productivity of methanol from H_2_/CO/CO_2_ depends on the junction between metal and oxide and is related to oxygen or anion vacancies. Recently, Tisseraud *et al*. explained the Cu-ZnO synergy in methanol thermo-synthesis in terms of the formation of the Cu_x_Zn_(1−x)_O_y_ interlayered phase between Cu and ZnO at 350 °C^45^. The role of oxygen vacancies in interacting with gaseous CO_2_ has been widely investigated for the gas-phase thermo-chemical reaction^6, 20^. Our (Cu,*Ni*)(O,S) closely related to the formed defective interlayered phase has successfully demonstrated the conversion of CO_2_ to EtOH even at the ambient environment.

The artificial synthesis of ethanol with the C-C bond formation is rare at the natural environment. The third proposal is given as: *The ethanol formation is related to the reaction between the pinned and the adsorbed oxygenates in considering the stereochemistry*. If all molecules on catalyst surface are held straight through the C-O bond pinning, they are all fixed and do not have the flexibility to form the C-C bonds.

This study demonstrates the conversion of dissolved CO_2_ to ethanol with the C-C bond formation over covellite-based (Cu,*M*)(O,S) oxysulfide catalysts with *M* = Ni, Sn, and Co in water. Several factors need to simultaneously operate together in order for this ambient conversion to occur, which are (1) the highly strained catalyst with anion vacancies to trap, pin, and weaken water and oxygenates, (2) the charge transport ability in semiconductor-type catalyst, and (3) the reaction between the pinned and the adsorbed C_1_ oxygenates to form the C_2_ ones. The amounts of ethanol derived from CO_2_ over a period of 20 h were 5.2, 1.5, 0.30, and 0 mg for (Cu,*Ni*)(O,S), (Cu,*Sn*)(O,S), (Cu,*Co*)(O,S), and Cu(O,S), respectively. Here the C_2_ oxygenate of ethanol with the C-C bond formation is formed from adsorbed CO_2_ and the pinned and active H_2_O at natural environment with inorganic catalysts. Therefore, CO_2_, water, and the suitable inorganic mineral are sufficient to execute the abiotic chemical synthesis of C_2_ oxygenate of ethanol.

## Electronic supplementary material

Supporting Information
