# Supplementary material for: Abiotic Synthesis with the C-C Bond Formation in Ethanol from CO2 over (Cu,M)(O,S) Catalysts with M = Ni, Sn, and Co
Source: Sci Rep. 2017 Aug 30;7:10094. doi: 10.1038/s41598-017-10705-3 (PMC5577111; doi:10.1038/s41598-017-10705-3)
Supplement: Supplementary file 1 — Supporting Information [file 41598_2017_10705_MOESM1_ESM.pdf]

Supporting Information

Abiotic Synthesis with the C-C Bond Formation in Ethanol from CO_2 over (Cu,*M*)(O,S) Catalysts with *M*= Ni, Sn, and Co

Xiaoyun Chen[1,2], Hairus Abdullah[1], Dong-Hau Kuo[1], Hsiu-Ni Huang[3], Cheng-Chung Fang[4]

[1] Department of Materials Science and Engineering, National Taiwan University of Science and Technology, Taipei 10607, Taiwan

[2] College of Material Engineering, Fujian Agriculture and Forestry University, Fuzhou 350002, China

[3] Instrument Center, Office of Research and Development, National Taiwan Normal University, Taipei City 11677, Taiwan

[4] Departments of Emergency Medicine, National Taiwan University Hospital and National Taiwan University College of Medicine, Taipei 100, Taiwan

Corresponding Author

*E-mail: dhkuo@mail.ntust.edu.tw

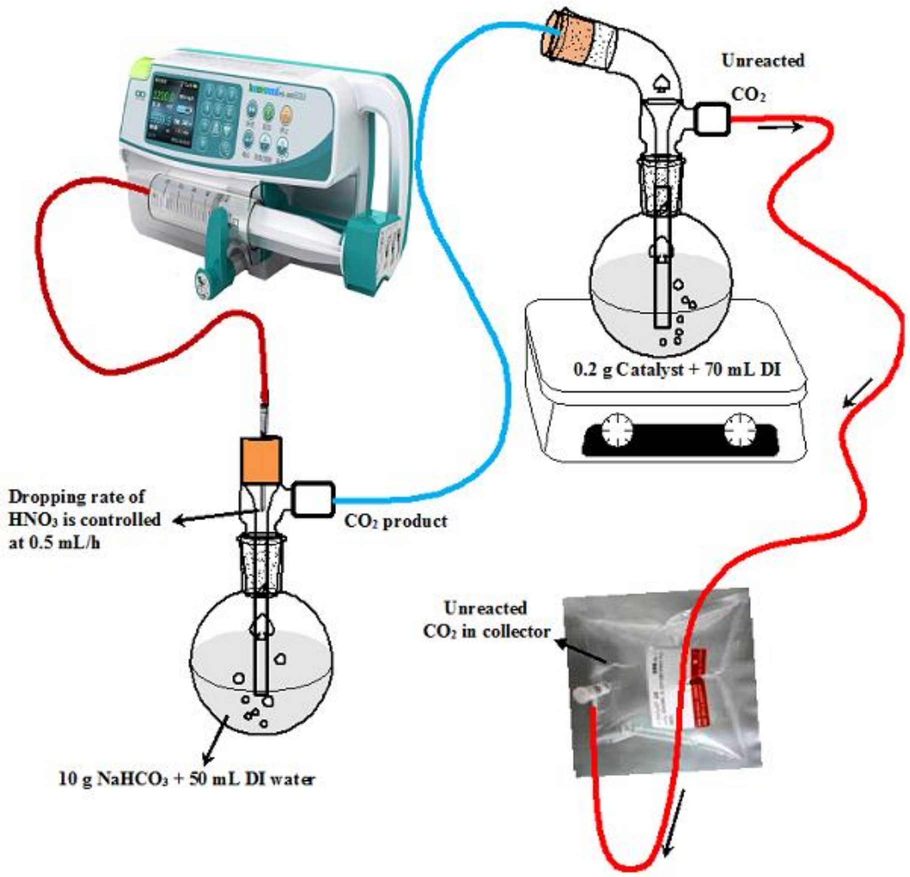

Figure S1 Schematic drawing of the setup for the CO_2-to-ethanol conversion experiment at ambient temperature and pressure using $NaHCO_3$ powder as a CO_2 source.

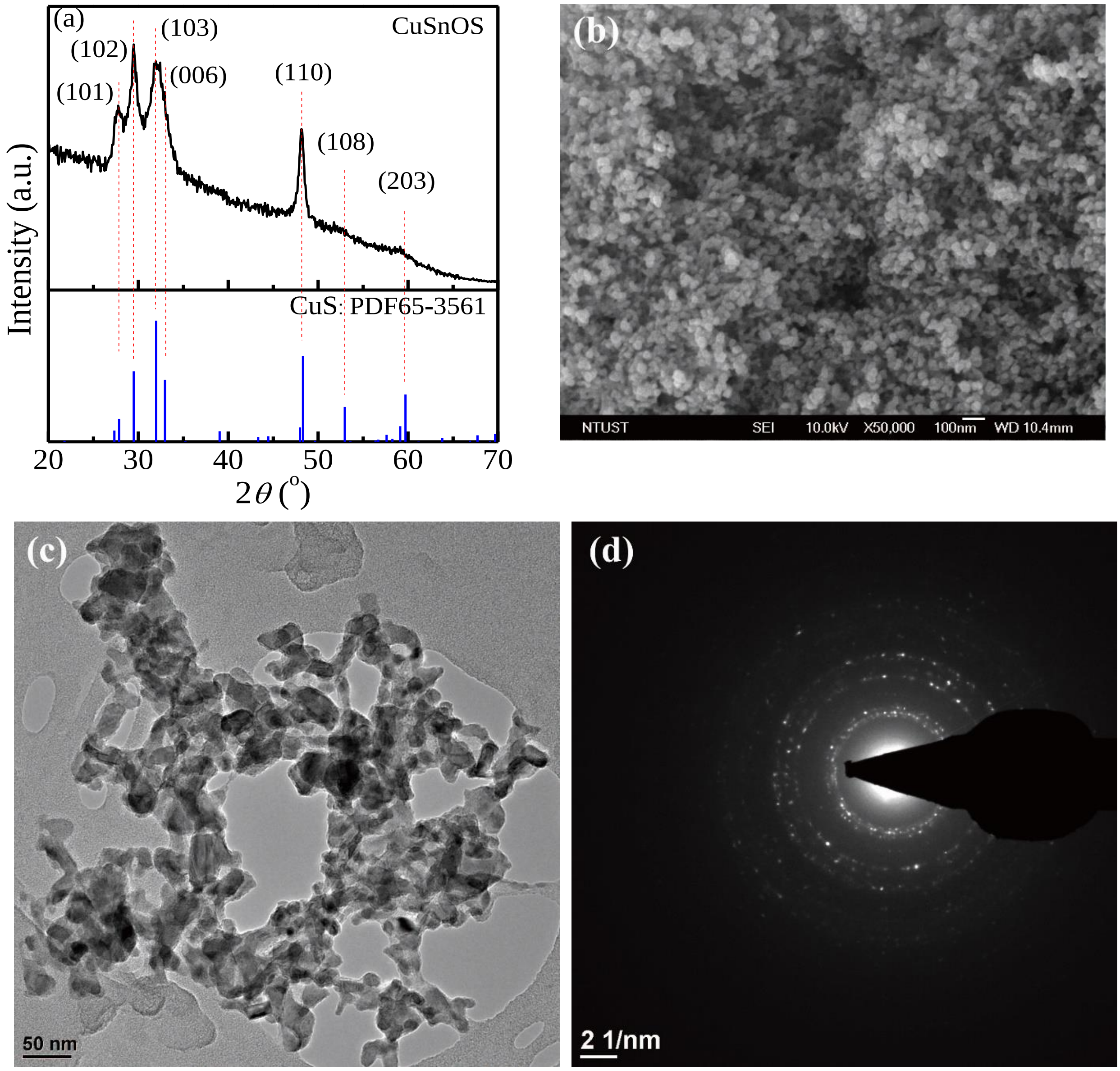

Figure S2. Microstructural and structural characterizations of (Cu,*Sn*)*(*O,S) catalyst: (a) XRD spectrum, (b) low-magnification SEM image, (c) high-magnification SEM image, (d) TEM image, (e) HR-TEM image, and (f) SAED pattern.

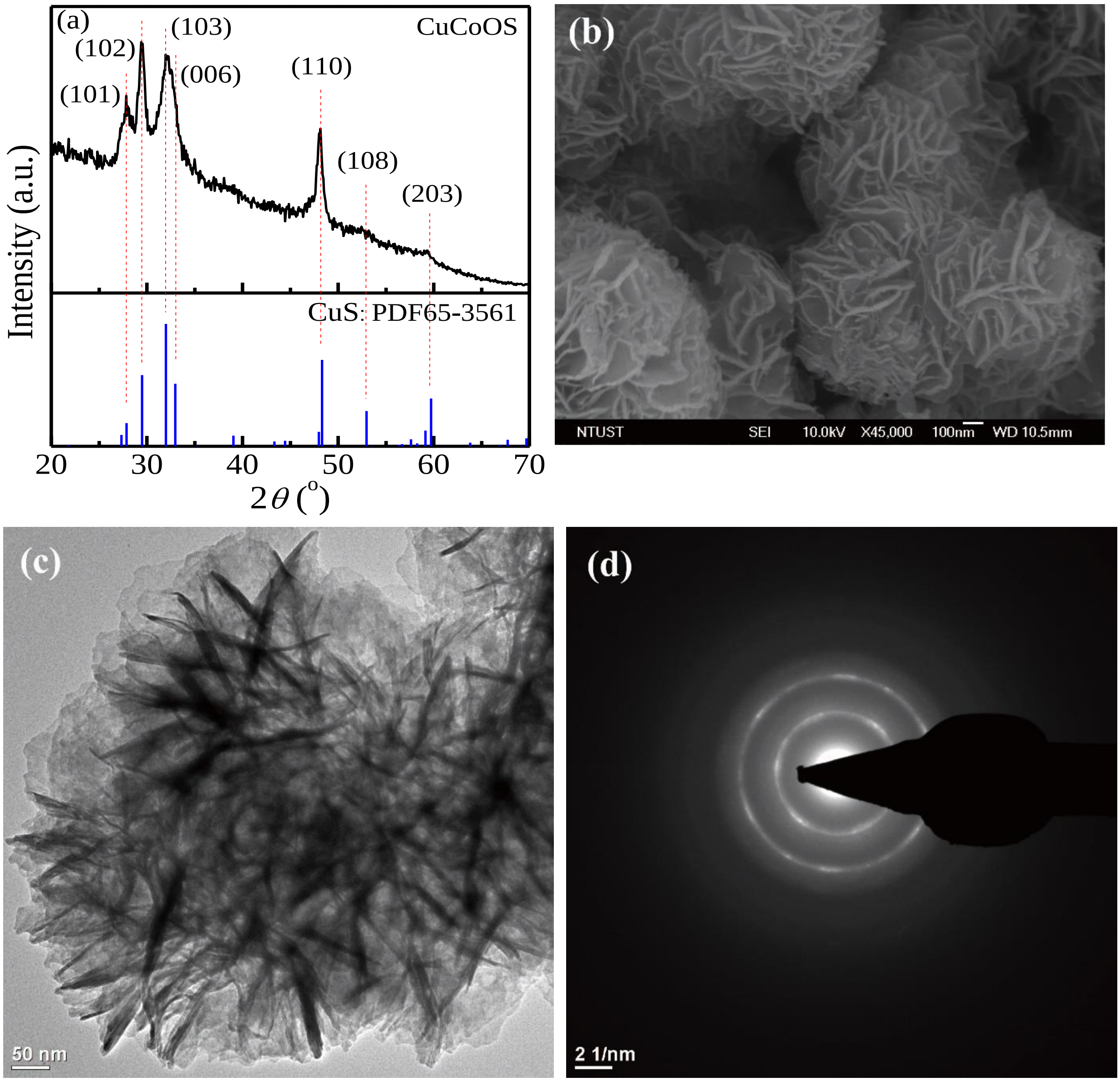

Figure S3. Microstructural and structural characterizations of (Cu,*Co*)(O,S) catalyst: (a) XRD spectrum, (b) low-magnification SEM image, (c) high-magnification SEM image, (d) TEM image, (e) HR-TEM image, and (f) SAED pattern.

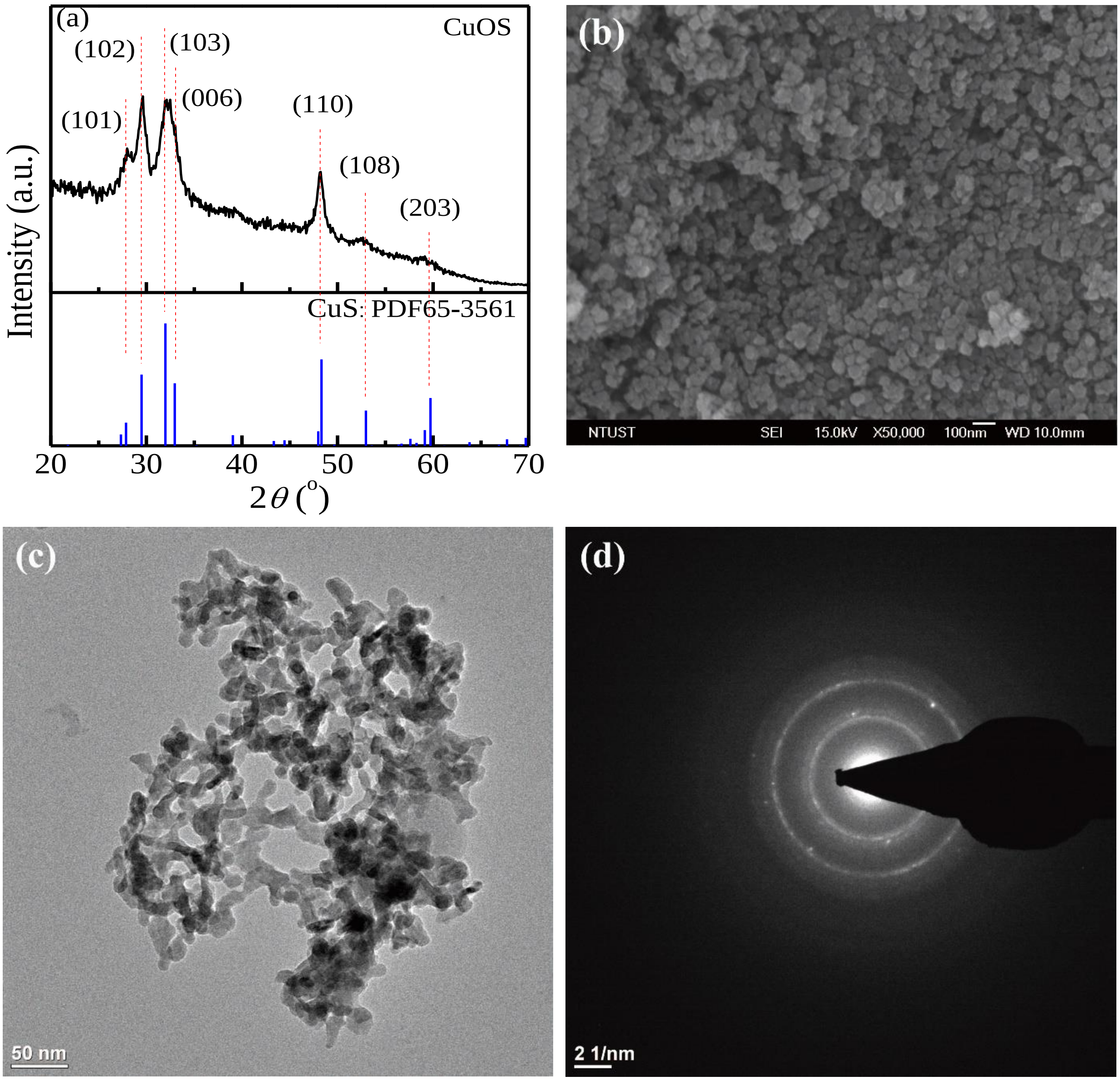

Figure S4. Microstructural and structural characterizations of Cu(O,S) catalyst: (a) XRD spectrum, (b) low-magnification SEM image, (c) high-magnification SEM image, (d) TEM image, (e) HR-TEM image, and (f) SAED pattern.

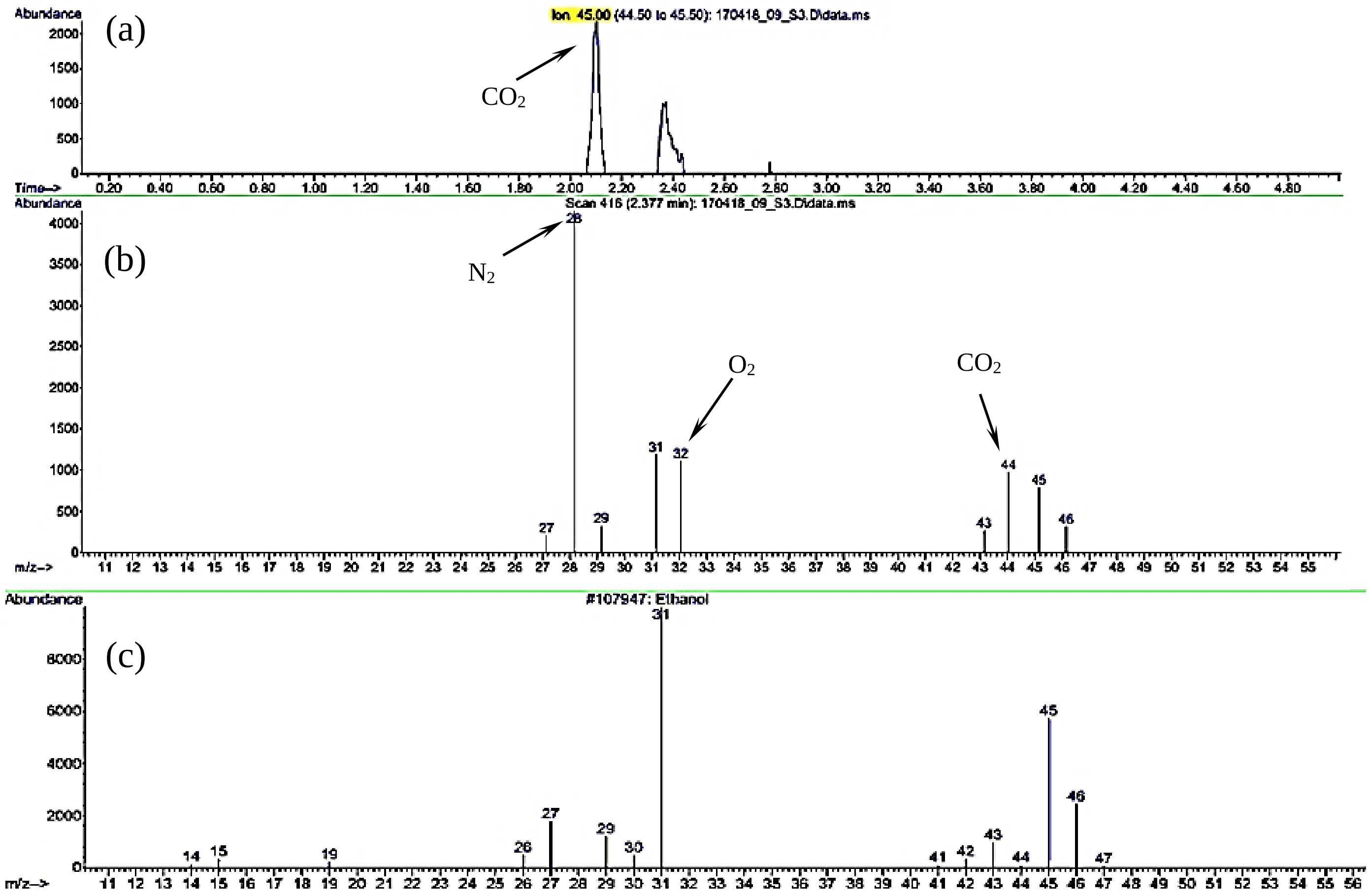

Figure S5 (a) Gas chromatogram of reaction solution catalyzed by (Cu,*Sn*)*(*O,S)with (b) the mass spectra in the retention time from 2.332 to 2.415 min. (c) The standard mass spectra of ethanol.

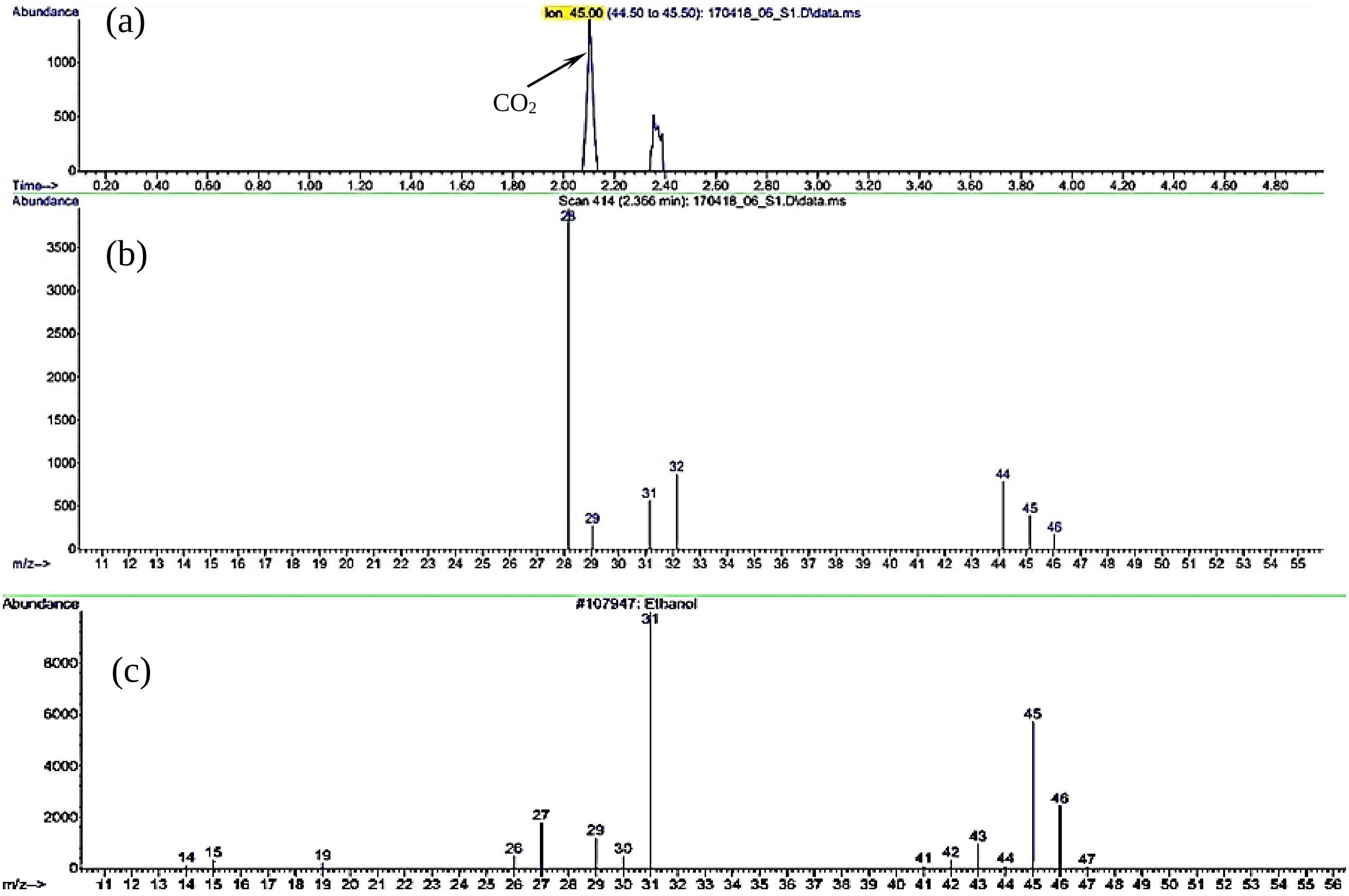

Figure S6 (a) Gas chromatogram of reaction solution catalyzed by (Cu,*Co*)*(*O,S) with (b) the mass spectra in the retention time from 2.332 to 2.415 min. (c) The standard mass spectra of ethanol.

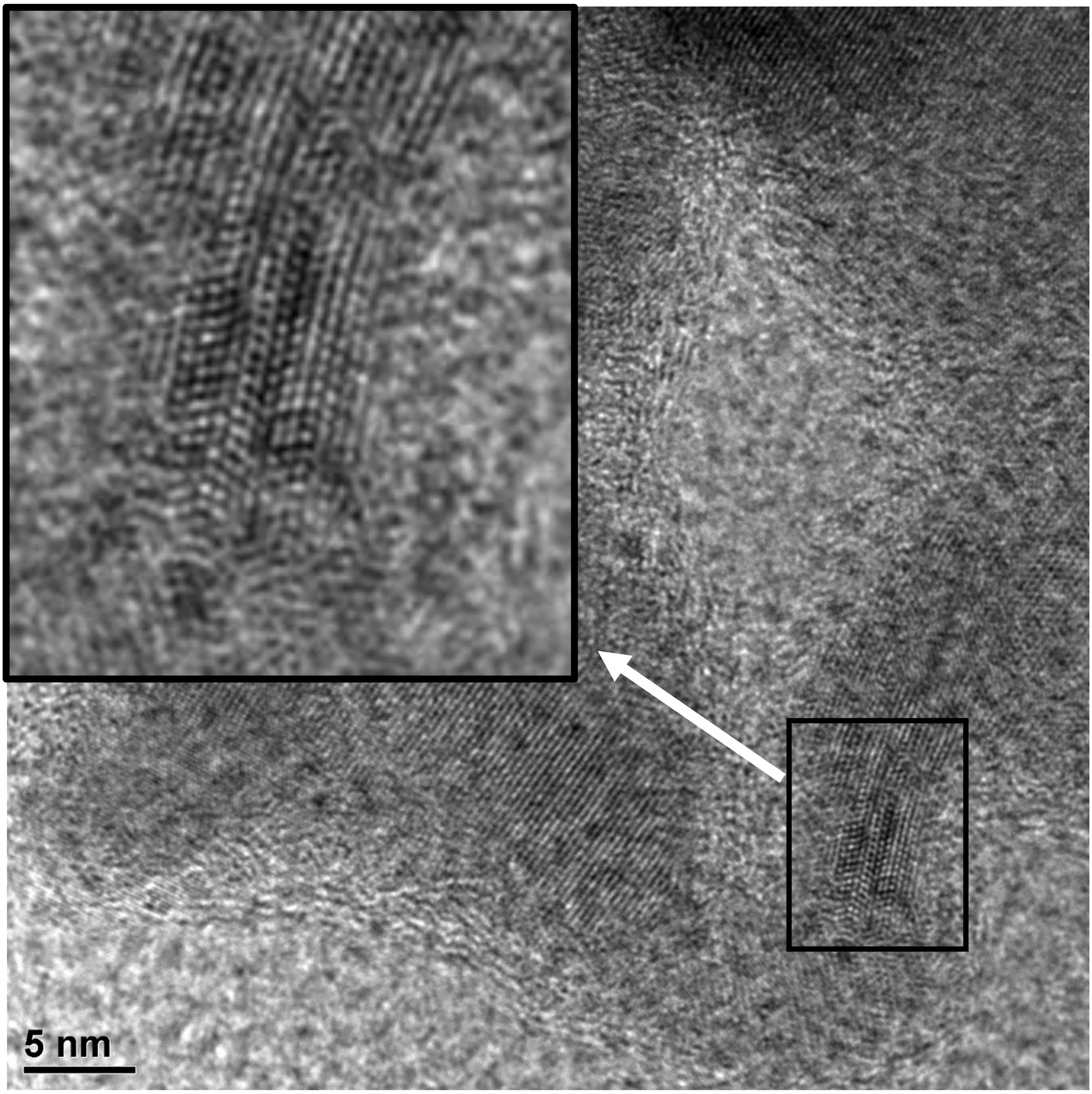

Figure S7 High resolution TEM image of (Cu,*Sn*)*(*O,S). The enlarged image inserted in figure is to show the bent and distorted lattice.

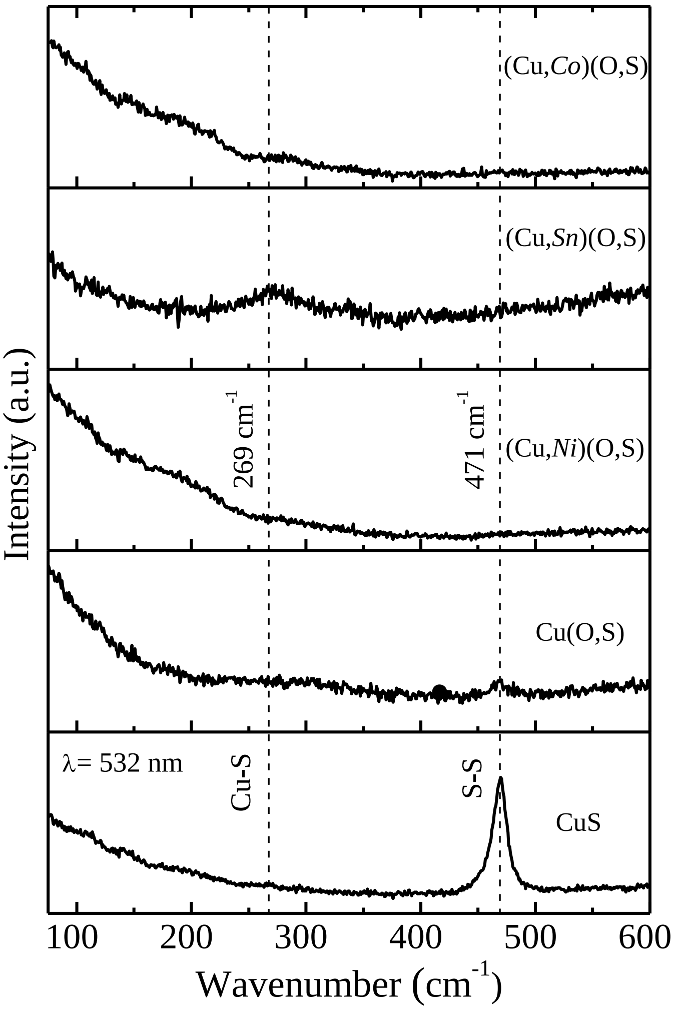

Figure S8 Raman spectra of (Cu,*M*)(O,S) catalysts with *M*= Ni, Sn, and Co. Self-synthesized Cu(O,S) and commercially available CuS powder were also tested.
